# Development of Colorimetric Reverse Transcription Loop-Mediated Isothermal Amplification Assay for Detecting Feline Coronavirus

**DOI:** 10.3390/ani12162075

**Published:** 2022-08-14

**Authors:** Witsanu Rapichai, Wichayet Saejung, Kotchaporn Khumtong, Chaiwat Boonkaewwan, Supansa Tuanthap, Peter A. Lieberzeit, Kiattawee Choowongkomon, Jatuporn Rattanasrisomporn

**Affiliations:** 1 Center for Advanced Studies for Agriculture and Food, Kasetsart University Institute for Advanced Studies, Kasetsart University, Bangkok 10900, Thailand; 2 Department of Companion Animal Clinical Sciences, Faculty of Veterinary Medicine, Kasetsart University, Bangkok 10900, Thailand; 3 Graduate Program in Animal Health and Biomedical Sciences, Faculty of Veterinary Medicine, Kasetsart University, Bangkok 10900, Thailand; 4 Akkhraratchakumari Veterinary College, Walailak University, Nakhon Si Thammarat 80161, Thailand; 5 Faculty of Veterinary Medicine, Rajamangala University of Technology Tawan-ok, Bangpra, Chonburi 20110, Thailand; 6 Department of Physical Chemistry, Faculty of Chemistry, University of Vienna, Waehringer Strasse 42, A-1090 Vienna, Austria; 7 Department of Biochemistry, Faculty of Science, Kasetsart University, Bangkok 10900, Thailand

**Keywords:** feline infectious peritonitis, reverse transcription loop-mediated isothermal amplification, molecular diagnosis

## Abstract

**Simple Summary:**

Feline coronavirus infecting domestic cats can cause feline infectious peritonitis (FIP), a fatal infectious disease. Several relevant clinical diagnoses and molecular methods are complicated and often ambiguous for veterinarians. In this work developed a rapid, sensitive, specific, and easy-to-visualize colorimetric reverse transcription loop-mediated isothermal amplification (RT-LAMP) assay with a novel LAMP primer set that has high specificity was developed using neutral red as an indicator dye. This proposed procedure could reliably detect FCoV RNA from effusion fluids comparable to the conventional PCR method. Considering these advantages, the RT-LAMP developed here has great potential on FIP-associated FCoV surveillance. Together with other sophisticated molecular diagnostic tools, this method can further be exploited in clinical laboratories to inspect suspected cats with effusive FIP.

**Abstract:**

Feline infectious peritonitis (FIP) is a worldwide fatal disease caused by a mutant feline coronavirus (FCoV). Simple and efficient molecular detection methods are needed. Here, sensitive, specific, rapid, and reliable colorimetric reverse transcription loop-mediated isothermal amplification (RT-LAMP) was developed to detect the ORF1a/1b gene of FCoV from cats with suspected FIP using neutral red as an indicator. Novel LAMP primers were specifically designed based on the gene of interest. The isothermal assay could visually detect FCoV at 58 °C for 50 min. The RT-LAMP assay was highly specific and had no cross-reactivity with other related feline viruses. The detection limit of FCoV detection by RT-LAMP was 20 fg/µL. A blind clinical test (*n* = 81) of the developed RT-LAMP procedure was in good agreement with the conventional PCR method. In the light of its performance specificity, sensitivity, and easy visualization, this neutral-red-based RT-LAMP approach would be a fruitful alternative molecular diagnostic tool for veterinary inspection of FCoV when combined with nucleotide sequencing or specific PCR to affirm the highly virulent FIP-associated FCoV.

## 1. Introduction

**Figure 1:**
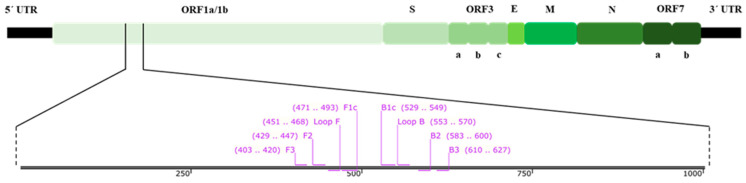
Localization of RT-LAMP primer set within ORF1a/1b sequence. The green shaded row presents the FCoV genome consisting of the 5′ untranslated region (5′ UTR), ORF1a/1b, spike (S), ORF3a, ORF3b, ORF3c, envelope (E), membrane (M), nucleocapsid (N), ORF7a, ORF7b, and 3′ untranslated region (3′ UTR).

Feline coronavirus (FCoV) is an enveloped, single-stranded, positive-sense RNA virus, classified as a member of the order *Nidovirales*, family *Coronaviridae*, genus *Alphacoronavirus*, subgenus *Tegacovirus*, species *Alphacoronavirus 1* [1,2,3]. It is closely related to human coronavirus NL63 and 229E, canine coronaviruses (CCVs), and porcine transmissible gastroenteritis virus (TGEV) [4,5]. Its genome of approximately 30 kb long is composed of two overlapping open reading frames (ORFs) at its 5’ end, ORF1a and ORF1b, which encode for non-structural functional proteins involved in viral RNA synthesis. The genome also contains other ORFs coding for structural proteins which are spike (*S*), envelope (*E*), membrane (*M*), and nucleocapsid (*N*) genes, along with those coding for the accessory proteins, ORF3abc and ORF7ab (Figure 1) [6,7,8].

FCoV is divided into serotypes I and II based on sequence analysis and antigenicity [9,10,11]. Type I FCoV is the largely predominant serotype causing infections in cats with a high worldwide prevalence in cats [12], whereas type II FCoV has arisen from recombination events of type I FCoV, and CCVs, yielding replacement of spike gene including a part of the adjoining ORF3 gene [13,14]. Also, both type I and II FCoV can cause the fatal disease of feline infectious peritonitis (FIP). In addition, FCoV can be classified into avirulent and virulent biotypes based on their pathobiology [15]. Avirulent strains of FCoV are capable of infecting the intestinal tract resulting in asymptomatic or mild digestive disease, so-called feline enteric coronavirus (FECV). It is widely accepted that the highly virulent biotype, referred to as feline infectious peritonitis virus (FIPV) develops from less virulent FCoV in individual cat through mutations in certain genes (spike glycoproteins and probably some accessory genes, 3c and 7b); the hypothesis of which is called internal mutation theory [15]. It was estimated to occur in approximately 5% of persistently infected cats [16,17,18,19]. FIPV is responsible for monocytes and macrophages infection leading to fatal immune-mediated disease [15,18,20,21].

FIP occurs as wet (effusive) and dry (non-effusive) forms [22]. An effusive FIP is characterized by accumulating thoracic or peritoneal effusions with clear, viscous, straw-yellow fluid. Cats with dry form FIP have been observed with multiple granulomas and serofibrinous lesions in the serosa [4]. Clinicopathological diagnosis via Rivalta’s test and effusion analysis are generally used, as well as veterinarian predictive interpretations from serum biochemical, cytological, and blood hematological parameters that are not conclusive [22,23]. Molecular methods for diagnosing cat-infected FCoV based on RNA amplification currently used are conventional reverse-transcriptase polymerase chain reaction (RT-PCR) [24,25], reverse-transcriptase quantitative PCR (RT-qPCR) [11,26,27,28,29,30], reverse transcriptase nested PCR (RT-nPCR) [31,32,33], and reverse-transcriptase loop-mediated isothermal amplification (RT-LAMP) [8,34,35] of which, the most common tests used nowadays are the RT-qPCR whereas the RT-nPCR has been used previously [36]. However, these PCR-based methods have several disadvantages. They are costly to be applicable in diagnostic laboratories and healthcare facilities, often not promptly available in resource-constrained areas for routine work, and need well-trained staffs [22]. Thus, loop-mediated isothermal amplification (LAMP) is suitable to be developed as an alternative for FCoV RNA detection in samples of cats suspected of having FIP due to its simplicity, convenience, sensitivity, specificity, efficiency, and cost-effectiveness. It is also commercially available for use in on-site veterinary practice. Here, we have attempted to develop a neutral red-based RT-LAMP (NR-based RT-LAMP) assay as a means for FCoV detection. 

## 2. Materials and Methods

### 2.1. Cell and Virus Propagation

Samples of the Crandell-Rees Feline Kidney (CRFK) cell line (CCL-94, ATCC) were cultured in Eagle’s Minimum Essential Medium (EMEM) (ATCC 30-2003, USA) supplemented with 10% horse serum (Invitrogen, Waltham, MA, USA) at 37 °C overnight in a 5% CO_2_ incubator. The growth medium was removed from 70–80% confluent monolayer cells, washed three times with 1X sterile PBS, and inoculated with feline infectious peritonitis virus (FIPV) (strain WSU 79-1146, ATCC VR-990) at 10^5^ TCID50/mL. The tissue culture infective dose (TCID50) was determined using the Reed-Muench method [37]. The cells were cultured continuously at 37 °C for 60 min under 5% CO_2_ incubation, with the added maintenance medium consisting of EMEM supplemented with 0.1 mM non-essential amino acids (Thermo Scientific, Wilmington, NC, USA), 100 U/mL penicillin, and 100 μg/mL streptomycin (Nacalai tesque, Kyoto, Japan). Fresh maintenance medium was changed daily. The inoculated cell cultures were observed for a cytopathic effect (CPE) for 3–5 days (characterized by cell fusion, cell rounding, shrinkage, and syncytial appearance), and cells partially detached from the flask. The cell cultures with CPE were harvested and collected culture supernatants were used for further viral RNA extraction viral RNA and PCR amplification with specific primer sets for FCoV identification. 

### 2.2. FCoV RNA Extraction

Total RNA from FCoV-infected CRFK culture supernatants were extracted using an E.Z.N.A. Viral RNA Kit (Omega Bio-Tek, Norcross, GA, USA) according to the manufacturer’s instruction, and was further used as templates for RT-LAMP optimization and as a positive control (PTC) throughout the experiments. FCoV cDNA synthesis was done using RNA extracted from culture supernatants as templates by a RevertAid First Strand cDNA Synthesis Kit (Thermo Scientific, Wilmington, NC, USA). Subsequently, the cDNA samples were checked for FCoV based on the nucleocapsid (*N*) gene using the F9N and R9N primers (Table 1) [18]. Cycling parameters were initial denaturation at 95 °C for 2 min, 35 cycles of denaturation at 94 °C for 30 s, annealing at 65 °C for 30 s, extension at 72 °C for 90 s, and final extension at 72 °C for 5 min. PCR amplicons of approximately 1087 bp were analyzed using agarose gel electrophoresis. The obtained FCoV RNA concentration was also determined using a NanoDrop spectrophotometer (Thermo Scientific, Wilmington, NC, USA) and prepared to the desired concentration with sterile RNase-free water.

### 2.3. RT-LAMP Primer Design

The two overlapping open reading frames (ORF), ORF1a/1b, of the polymerase (*pol*) gene have been reported to be the most highly conserved sequence for coronavirus [38]. Therefore, the ORF1a/1b sequences of FCoV serotype II strains from the GenBank database with accession numbers of NC_002306, AY994055, KC461235, KC461236, KC461237, JQ408980, JQ408981, DQ286389, JN634064, GQ152141, MT239439, and MW030109 were used to design the LAMP primer using the Primer Explorer V5 software (https://primerexplorer.jp/lampv5e/; accessed on 18 October 2021). The designed primers were checked for their specificity with the sequences of feline coronavirus strain FCoV C1Je (DQ848678), feline infectious peritonitis virus (AY994055), feline immunodeficiency virus (M25381), feline leukemia virus Kawakami-Theilen strain KT-FeLV-UCD-1 (MT129531), feline herpesvirus 1 strain C-27 (NC_013590), feline calicivirus SH (KP987265), and feline panleukopenia virus isolate GX01 (MG924893) using the Primer-BLAST software [39]. Moreover, different feline housekeeping genes including the followings: *S14* housekeeping gene (DQ154258), beta-actin (*ACTB*), beta-2-microglobulin (*B2M*), beta-glucuronidase (*GUSB*), hydroxymethylbilane synthase (*HMBS*), tyrosine 3-monooxygenase/tryptophan 5-monooxygenase activation protein-zeta polypeptide (*YWHAZ*), and ribosomal proteins L17 (*RPL17*), L30 (*RPL30*), S7 (*RPS7*), S9 (*RPS9*), and S19 (*RPS19*) were also checked with the same software. The LAMP primer set, comprised two outer primers (F3 and B3), two inner primers (FIP and BIP), and two loop primers (Loop F and Loop B), which were capable of recognizing eight distinct regions on their 225 bp target sequence. They were synthesized by Macrogen (Seoul, South Korea). Figure 1 shows the FCoV genomic structure and LAMP primer position, while Table 1 shows the designed LAMP primer sequences.

### 2.4. RT-LAMP Reaction and Optimization

A master mix of NR-based RT-LAMP reaction was prepared in a total volume of 25 μL by adding the following ingredients: 2.5 μL 10X ammonium sulfate buffer (100 mM (NH_4_)_2_SO_4_, 100 mM KCl, and 10% Tween 20 pH 8.8), 6 μL 25 mM MgCl_2_, 3.5 μL 10 mM dNTP solution mixture (Yeastern Biotech, New Taipei City, Taiwan), 1.25 μL 20X LAMP primer mix (1.6 µM each of FIP and BIP, 0.2 µM each of F3 and B3 primers, and 0.4 µM each of Loop F and Loop B primers), 1 µL 40 U/µL RiboLock RNase inhibitor (Thermo Scientific, Wilmington, NC, USA), 1 μL 2.5 mM Neutral Red (NR) (Invitrogen, Waltham, MA, USA), 1 μL 8 U/μL *Bsm* DNA polymerase, Large Fragment (Thermo Scientific, Wilmington, NC, USA), 0.5 μL 200 U/μL RevertAid reverse transcriptase (Thermo Scientific, Wilmington, NC, USA), 7.25 μL RNase-free water (Apsalagen, Thailand), and 1 μL 10 ng RNA sample. RT-LAMP amplification reaction was performed using a thermal cycler (Bio-Rad, Hercules, CA, USA) at 58 °C for 50 min, followed by inactivation at 80 °C for 5 min. RNase-free water was used as a negative control (NTC) in all experiments. To avoid unknown contamination, RT-LAMP master mixes were prepared in a cleaned PCR cabinet.

To determine the optimum conditions for RT-LAMP, the following essential parameters were optimized as follows: amplification temperatures (50, 50.7, 51.9, 53.8, 56.1, 58, 59.2, 60, 61, 61.7, 62.8, 64.4, 66.5, 68.2, 69.4 and 70 °C), amplification times (10, 20, 30, 40, 50, and 60 min), MgCl_2_ concentrations (2, 4, 6, 8, and 10 mM), dNTP concentrations (0.6, 1.0, 1.4, 1.8, and 2.2 mM), and a primer requirement test.

### 2.5. RT-LAMP Product Analysis

In this colorimetric detection, a pink color indicated a positive reaction and a yellow color indicated a negative one. After amplification, the LAMP reactions were photographed for documentation using a smartphone camera and the original images were adjusted for clear color based on the VIVID mode. For visualization detection, a LAMP amplicon was confirmed by 1.5% (*w*/*v*) agarose gel electrophoresis. An agarose gel powder was prepared with 1X TAE buffer (2 M Tris-Acetate, 0.05 M EDTA), pH 8.3 (Omega Bio-Tek, Norcross, GA, USA). Ten microliters of each LAMP amplicon or 100 bp DNA Ladder (Yeastern Biotech, New Taipei City, Taiwan) mixed with 1 µL 6X loading dye were run on the gel at 80 V for 45 min. The gel was further visualized under a BluPAD LED Transilluminator (BIO-HELIX, New Taipei City, Taiwan).

### 2.6. RT-LAMP Specificity Analysis

To test the specificity of the RT-LAMP reaction, viral genomic materials, DNA or RNA, were extracted from clinical samples infected with different feline viruses, consisting of feline coronavirus (FCoV), feline immunodeficiency virus (FIV), feline leukemia virus (FeLV), feline herpesvirus (FHV), feline calicivirus (FCV), and feline panleukopenia virus (FPLV). These were amplified with LAMP primers. Each reaction was performed under the optimum RT-LAMP conditions. RNA extracted from uninfected CRFK cells, a representative feline cell, and buffy coats, a representative feline white blood cell, were also tested as internal controls. RNase-free water was used as the negative control (NTC) and the FCoV strain WSU 79-1146 RNA extracted from the cell culture was used as the positive control. 

To confirm the specificity of the RT-LAMP primers, the smallest band of positive LAMP product was excised from agarose gel and purified using a GeneJET Gel Extraction Kit (Thermo Scientific, Wilmington, NC, USA). Using the primer pairs of F2 and B2 as the sequencing primers (Table 1), the purified DNA was submitted to Macrogen (Seoul, South Korea) for nucleotide sequencing. Sequence similarity analysis was performed by aligning an obtained sequence of the ORF1ab fragment with those in GenBank database (NCBI) using the BLAST search program.

### 2.7. Reverse Transcription PCR (RT-PCR)

To compare the sensitivity of RT-LAMP for FCoV detection, one-step reverse transcription PCR (RT-PCR) was performed using the F7 and R7Sc primers (Table 1), which were reported to be FCoV type II-specific primers designed from the ORF3 region [18]. The RT-PCR reaction of 25 µl was prepared using a Deoxy^+^ OneStep RT-PCR kit (Yeastern Biotech, New Taipei City, Taiwan) by adding 1 µL each of 10-fold diluted FCoV RNA, 1.5 µL 0.6 µM of each primer, and 8.5 µL RNase-free water into the 12.5 µL 2X OneStep RT-PCR premix. The reverse transcription was performed at 42 °C for 60 min and the reaction inactivated at 70 °C for 7 min. The PCR parameters were 35 cycles of denaturation at 94 °C for 30 s, annealing at 65 °C for 30 s, and extension at 72 °C for 1.5 min, followed by a final extension at 72 °C for 5 min. An amplicon of approximately 1000 bp was then analyzed in 1.5% (*w*/*v*) agarose gel. 

### 2.8. Comparison of RT-LAMP and RT-PCR on Sensitivity for FCoV Detection

The sensitivity of the RT-LAMP procedure was determined according to the previously reported method [40,41]. Briefly, it was tested by determining 10-fold serially diluted FCoV RNA in the concentrations of 2000 ng/µL, 200 ng/µL, 20 ng/µL, 2 ng/µL, 200 pg/µL, 20 pg/µL, 2 pg/µL, 200 fg/µL, 20 fg/µL, 2 fg/µL, 200 ag/µL, 20 ag/µL, and 2 ag/µL. The sensitivity was also compared with that of the conventional RT-PCR with the same serial dilutions, based on the amplified ORF3 gene using the F7 and R7Sc primers [18]. Each LAMP reaction was amplified under optimum conditions. The detection limit was defined as the lowest copy number of the amplicon positively detected by both pink color appearance and agarose gel electrophoresis. RNase-free water was used as the negative control (NTC). To investigate the detection limit in terms of viral copy molecules per microliter, the lowest FCoV RNA concentration detected was calculated using the following equation [42]:

$$Y\;(\mathrm{copies}/µL)=\frac{X\;(g/µL)\;\times \;6{.022\;\times \;10}^{23}}{Z\;(\mathrm{bp})\;\times \;340}$$

where Y is the copy number of viral molecules, X is the nucleotide concentration, and Z is the nucleotide length.

### 2.9. Clinical Samples Test Using RT-LAMP Assay 

To apply the RT-LAMP procedure at the clinical level, 81 samples of clinical specimens (effusion fluid from thoracic and/or abdominal cavity of cats suspected to be FIP) were collected from at least four of the following: abnormal clinical signs (with abdominal enlargement), hematological and biochemical profile changes, diagnostic imaging (X-rays or ultrasound examinations) showing effusion fluid accumulation within the thoracic and/or abdominal cavity, positive Rivalta’s test, or positive PCR result (FCoV detection). FCoV RNA was prepared according to the previously reported method [18]. In brief, body fluid was vigorously vortexed, aliquoted to 300 µL, mixed with 1X phosphate buffer saline, pH 8.3 at the ratio of 1:3, and centrifuged at 14,000 rpm for 5 min. Then, the supernatant was extracted for RNA, which was subsequently used as a template for RT-LAMP examination in a blind manner. FCoV RNA obtained from the cell culture was used as the positive control. RNase-free water was used as the negative control (NTC).

### 2.10. Statistical Analysis

A two-by-two table was created for the RT-LAMP and conventional PCR procedures for detecting FCoV. Comparative values of sensitivity and specificity were calculated as percentages in order to assess the efficiency of RT-LAMP for FCoV detection. The Cohen’s kappa coefficient (ĸ) value was calculated and evaluated in agreement with conventional PCR. Based on the ĸ result, a value of less than 0.40 indicated a weak level of agreement, 0.41–0.6 indicated a good level of agreement, 0.61–0.80 indicated a substantial level of agreement, and 0.81–1.00 indicated a perfect level of agreement [43,44,45]. 

## 3. Results

### 3.1. Optimization of RT-LAMP Reaction

To detect FCoV based on the NR-based RT-LAMP reaction, the positive reaction was naked-eye visualized using NR dye as an indicator, yielding a color change from yellow to pink, corresponding to the number of generated protons as by product that was released during the LAMP process. The optimum temperature of the colorimetric RT-LAMP assay was determined. As shown in Figure S1, at 60 min of amplification with a gradient temperature in the range of 50–70 °C, the optimum temperature was 58 °C (showing the most intense pink color), which was used in all subsequent experiments. At the optimum temperature, the LAMP reactions were amplified for a range of times from 10 to 60 min. Figure S2 shows that amplification reaction appeared as a pink color in as short as 30 min. With naked-eye observation, at 40–60 min there was an apparently clear pink color that was most intense at 50 min. Hence, 50 min was chosen for further experiments. To determine the effect of the MgCl_2_ concentration, different concentrations (2, 4, 6, 8, and 10 mM) were tested. Figure S3 shows that at 6 mM MgCl_2_, a pink color was clearly observed. The effect of dNTPs was investigated by titrating with varying concentrations (0.6, 1.0, 1.4, 1.8, and 2.2 mM). Figure S4 indicates that dNTPs at the concentration of 1.4 mM showed the clearest clear pink color. To test the importance of the primers, either the inner primers (FIP and BIP) or the loop primers (Loop F and Loop B) was removed one reaction at the time. Figure S5 shows that all six primers were essential for amplifying FCoV RNA using the NR-based RT-LAMP system. In summary, the optimized parameters for the developed NR-based RT-LAMP assay for FCoV detection were: 6 mM MgCl_2_, 1.4 mM dNTPs, 1X all primers, 4 U/µL reverse transcriptase activity, and 0.32 U/µL *Bsm* DNA polymerase, and amplification at 58 °C for 50 min.

### 3.2. Specificity of Developed RT-LAMP for Detection of FCoV

**Figure 2:**
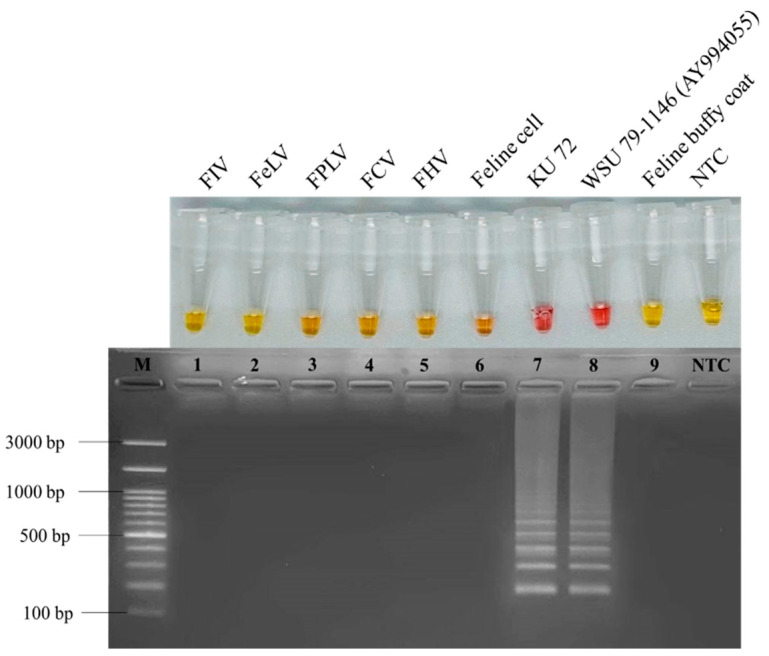
Specificity of RT-LAMP primers for FCoV detection. Different feline viruses, feline cells, and feline buffy coats amplified using the RT-LAMP primers were detected based on NR indicator dye (upper row) and by agarose gel electrophoresis (lower row). The positive control, WSU 79-1146 (AY994055), was pink color and the negative control (NTC) was yellow color. Lane M, 100 bp Ladder DNA Marker III (Yeastern Biotech, New Taipei City, Taiwan); lane 1-7, FIV, FeLV, FPLV, FCV, FHV, feline cell, FCoV effusion fluid sample no. KU72, respectively; lane 8, PTC, positive control (FIPV strain WSU79-1146, AY994055); lane 9, feline buffy coat; lane NTC, negative control.

The RT-LAMP primer set designed here was based on the conserved ORF1a/1b fragment of FCoV. It was cross-tested with reference sequences of feline viruses consisting of FIV (M25381), FeLV (MT129531), FPLV (MG924893), FCV (KP987265), FHV (NC_013590), FCoV (DQ848678 and AY994055). For internal controls, uninfected CRFK cells and buffy coats were also tested to evaluate potential feline housekeeping genes as follows: *S14* housekeeping gene (DQ154258), beta-actin (*ACTB*), beta-2-microglobulin (*B2M*), beta-glucuronidase (*GUSB*), hydroxymethylbilane synthase (*HMBS*), tyrosine 3-monooxygenase/tryptophan 5-monooxygenase activation protein-zeta polypeptide (*YWHAZ*), and ribosomal proteins L17 (*RPL17*), L30 (*RPL30*), S7 (*RPS7*), S9 (*RPS9*) and S19 (*RPS19*). The results of Primer-BLAST analysis revealed primer specificity only with FCoV. To evaluate the reaction specificity, cross-reactivity of the RT-LAMP primers was determined by amplifying genomic materials from the clinical samples, CRFK cells, and feline buffy coats. The FCoV clinical sample no. KU72 taken from an FIP cat with symptoms of jaundice and ascites with clear, viscous, and straw-yellow body effusion fluid was chosen for the specificity test. This sample had earlier been reported to contain FCoV by analysing the nucleotide sequence available in the GenBank database (accession number: MW545843, MW545892, MW545901, MW545910, and MW558578) [18]. As seen in Figure 2, only FCoV field sample no. KU72 showed pink color corresponding to that of the positive control, WSU 79-1146, while other samples gave yellow color of negative reactions. When run in agarose gel to confirm amplification products, only sample no. KU72 showed DNA band pattern as same as those of positive control. The results showed that the developed RT-LAMP primers produced no cross-reactivity among the tested feline viruses, indicating high specificity for FCoV detection. To verify the identity of the positive FCoV product, the lowest DNA band in lane 7 was purified from the gel and sent for sequencing using the Sanger method. The sequencing results showed approximately 99.41% identity (data not shown) with the ORF1ab sequence of the feline coronavirus strain DF-2 (DQ286389), indicating the specificity of the designed RT-LAMP primers for FCoV detection. 

### 3.3. Comparative Sensitivity of RT-LAMP and RT-PCR for FCoV Detection

**Figure 3:**
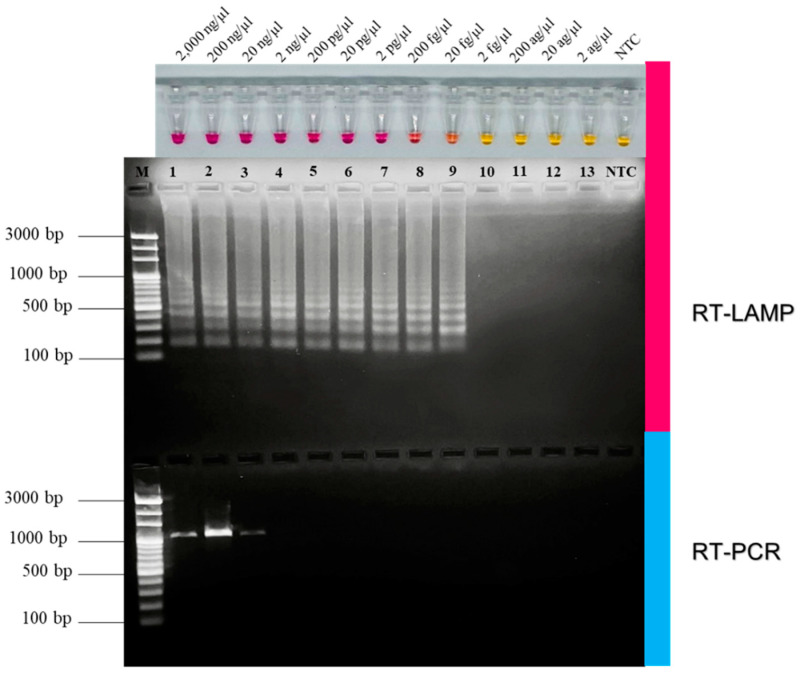
Comparative sensitivity of RT-LAMP and RT-PCR for FCoV detection. Pink bar shows the sensitivity of RT-LAMP detected by NR-based method confirmed by agarose gel electrophoresis. Blue bar shows the sensitivity of RT-PCR. Lane M, 100 bp Ladder DNA Marker III (Yeastern Biotech, New Taipei City, Taiwan), lane 1-13, amplification products of 10-fold serially diluted FCoV RNA at 2000 ng/µL, 200 ng/µL, 20 ng/µL, 2 ng/µL, 200 pg/µL, 20 pg/µL, 2 pg/µL, 200 fg/µL, 20 fg/µL, 2 fg/µL, 200 ag/µL, 20 ag/µL, and 2 ag/µL, respectively. Lane NTC, negative control.

The extracted FCoV RNA was 10-fold serially diluted with RNase-free water. The analytical efficiency of RT-LAMP was determined and compared with conventional RT-PCR with the same dilution series. As seen in Figure 3, the RT-LAMP positive reaction appeared pink and could detect FCoV RNA as low as 20 fg/µL, corresponding to 1.5 × 10^5^ copy molecules/µL. The pink RT-LAMP color results agreed well with that of the ladder-like DNA band pattern from the agarose gel electrophoresis. RT-PCR was able to detect the single DNA band of about 1000 bp as low as 20 ng/µL, corresponding to 3.5 × 10^10^ copy molecules/µL. As summarized in Table 2, these results indicated that the detection limit of our developed RT-LAMP was 10^6^ folds lower than that of RT-PCR. Additionally, the amplification time for RT-LAMP (50 min) was faster than that of RT-PCR (~3 h) indicating the high efficiency of NR-based RT-LAMP for FCoV diagnosis. 

### 3.4. Evaluation of RT-LAMP with Clinical Samples

To confirm the applicability of the RT-LAMP assay to detecting FCoV from effusion fluids in this context, a blind test with 81 RNA samples extracted from effusion fluids were determined. The RT-LAMP results were compared to those using the conventional PCR method. The diagnostic performance of this approach was estimated by statistical analysis using conventional RT-PCR as reference means. Of the 81 extracted clinical samples, 61 samples yielded positive results, one of which was negative with the conventional PCR. Four samples of negative results with RT-LAMP gave positive results when detected using conventional RT-PCR (Table S1). The comparative sensitivity and specificity of RT-LAMP with those of conventional PCR were 94% (with confidence interval (CIs), 61.1–100.0) and 94.12% (95% CIs, 55.8–100.0), respectively. The kappa coefficient (ĸ) value of 0.79, corresponding to the substantial level of agreement, by this RT-LAMP developed here clearly proves that the method could very well be used alternatively for FCoV detection along with conventional PCR (Table 3). 

## 4. Discussion

FCoV infection in cats is commonly found with mild enteritis. If natural mutation occurs, FCoV becomes a highly pathogenic strain, causing severe systematic inflammation [46]. Biotypic switching from FECV to FIPV, a lethal FCoV, has been reported to be approximately 5% in infected domestic cats [9,46]. Thus, it is reasonable that the difficulty in inspecting FIP cats due to their pathotypic switching might involve certain accessory genes (ORF3 and ORF7) and nucleocapsid gene mutations [18]. Several researchers have attempted to develop accurate molecular diagnostic tools to entail efficient diagnostic strategies in FIPV-infected cats [7,47,48,49]. It was found from our previous work that the sequence of Thai FCoV ORF3 gene contained hypervariability; therefore, more in-depth data are required in order to identify Thai FCoV through ORF3 gene [18]. 

LAMP technique developed by Notomi and colleagues for hepatitis B virus detection [50] has been used to overcome the limitations of PCR method [51]. This novel approach is practically performed under isothermal conditions without using any advanced equipment. Therefore, this method is applicable on-site as a simple, sensitive, and rapid diagnostic means for coronavirus infection in animals [52,53,54], humans [55,56,57,58], and zoonotic SARS-CoV-2 transmission from cats to humans [59]. LAMP reaction is capable of amplifying nucleic acid utilizing either *Bst* or *Bsm* DNA polymerase with strand displacement activity [51]. The amplification is carried out by a set of six primers recognizing eight distinct regions of the targeted gene [60], yielding a large amount of cauliflower-like DNA structures and insoluble pyrophosphate as a by-product [51]. LAMP amplicons can be detected by intercalating fluorescent dye [61], the turbidity of white Mg-pyrophosphate precipitate [62], agarose gel electrophoresis [63], and visualization using a metal-sensitive dye such as calcein [63] or hydroxynaphthol blue (HNB) [64]. It can also be observed using the color change of pH-sensitive dyes such as xylenol orange [65], phenol red [66], and neutral red (NR) [67], as a result of the generation of protons from the polymerization reaction [68]. When compared to the methods using intercalating fluorescent dye and metal-sensitive dye, the NR-based detection system has two important advantages, that is distinct contrast of color change of positive (pink color) and negative (yellow color) reaction. While violet changed to blue for HNB [55,64], dark blue changed to light blue for malachite green [69], and dark yellow changed to yellow for calcein [63], which were less discernable color changes based on their effects, disadvantage for these dyes [70]. Another advantage of the NR dye is its ability to easily visualize the color change of the reaction by naked eye without the risk of post-contamination from re-opening the tube [67,71,72]. This advantage is due to the release of hydrogen ions in isothermal amplification, causing NR to be protonated and changed to cationic form, affecting its contrast color shifts from yellow to pink [73]. Thus, it is most reasonable to utilize NR as an appropriate pH-sensitive dye for distinct positive and negative differentiation in LAMP assay [66] for detecting patulin-producing *Penicillium* species [67], species-specific of *P. expansum* [71], *Photobacterium* spp. [74], and African swine fever virus (ASFV) [72]. 

In this work, a set of six LAMP primers was designed based on the highly conserved ORF1a/1b gene of FCoV which recognize eight distinct regions. Colorimetric reaction assay was optimized with FCoV RNA as a template and positive reaction was detected via the appearance of pink color using NR as an indicator. The yellow/pink color change was clearly detectable after 40 min. 

Recently, several studies have developed rapid diagnostic RT-LAMP for feline coronavirus based on the 3′UTR region [34,35] and membrane gene [8]. While 6 primers were commonly used in other works, Techangamsuwan et al. reported the use of only 4 primers, without loop primers, for FCoV detection [34]. In order to examine the right set of FCoV primers most suitable for our systems, the roles of inner primers and loop primers were determined. Our proposed assay proved that a set of 6 primers could recognize eight distinct regions, and could effectively and specifically amplify the ORF1a/1b gene. 

Regarding specificity, this RT-LAMP primer set for FCoV was exploited for complementarity evaluation with reference sequences of FCoV, FIV, FeLV, FPLV, FHV, and FCV. In our lab, 11 feline reference genes were included to confirm cross-reactivity of LAMP primers. The genes examined were *S14* housekeeping gene (DQ154258), beta-actin (*ACTB*), beta-2-microglobulin (*B2M*), beta-glucuronidase (*GUSB*), hydroxymethylbilane synthase (*HMBS*), tyrosine 3-monooxygenase/tryptophan 5-monooxygenase activation protein-zeta polypeptide (*YWHAZ*), and ribosomal proteins L17 (*RPL17*), L30 (*RPL30*), S7 (*RPS7*), S9 (*RPS9*) and S19 (*RPS19*) [75,76]. Moreover, uninfected CRFK cells and feline buffy coats were employed as representatives of feline cells and feline white blood cells for internal controls. In-silico primer analysis was, as expected, highly specific for FCoV since there was no cross-reactivity with other feline viruses, especially with feline housekeeping genes. FCoV specificity could be confirmed by nucleotide sequencing of the smallest DNA band of LAMP product purified from the gel [67,71]. The sequencing result showed that the FCoV specificity was identical to that of feline coronavirus strain DF-2, DQ286389, confirming that our newly designed primers have enabled the reaction to achieved high specificity and were suitable for use at the clinical level. However, in previous published studies on RT-LAMP for the detection of feline coronavirus, the detection limits for their developed assays were not mentioned [8,34,35]. There was certain work involving a colorimetric LAMP assay using neutral red developed to detect ASFV which was allowed to detect p72 gene within recombinant plasmid pUC57 as low as 10 copies per reaction by either Tp analysis, direct naked-eye observation, or the agar gel-based electrophoresis [72]. As for the NR-based RT-LAMP FCoV assay developed here, the detection limit was determined from the lowest viral copy detected and compared to that of RT-PCR as a standard molecular method in an animal hospitals. In our RT-LAMP assay, the detection limit was found to be 20 fg/µL (1.5 × 10^5^ copy molecules/µL), which was approximately 10^6^ folds lower than that of RT-PCR. This finding indicated that our approach was more sensitive than RT-PCR. Even at the lowest concentration detected, a clear distinct result could easily be noticed either by naked-eye observation or by agarose gel electrophoresis, consistent with the colorimetric assay detecting ASFV mentioned above [72]. 

To validate the RT-LAMP with clinical specimens, the efficacy of RT-LAMP was compared with that of conventional PCR through blind testing using clinical samples of 81 effusion fluids, including positive and negative samples. The results demonstrated that our developed RT-LAMP assay had high potential and efficiency. As for one sample that was negative in type II specific conventional PCR but positive with RT-LAMP, it might be from a cat infected with type I FCoV. Even though RT-PCR is a rather conventional method, it is still sophisticated enough for use as a standard method for detecting FCoV RNA in different clinical samples to inspect FIP disease. The well-known disadvantages of RT-PCR; briefly, time-consuming, less sensitivity, post-PCR handling leading to carryover contaminations, and requirements for thermal cycler have been solved by RT-qPCR technology [22,77]. However, real-time RT-PCR needs a high-cost instrument and restricts its use in laboratories with good financial support. It is then a necessity for the researchers who work with limited funding that the result of the RT-LAMP assay be compared with those of conventional instrument. 

Hence, the NR-based RT-LAMP presented here provides a developed method as a proof-of-concept assay. Even though, this RT-LAMP requires RNA extraction or cDNA synthesis, with its sensitivity, specificity, and the ease of visualization of LAMP products, the developed RT-LAMP assay could be beneficial for veterinarians as an alternative, molecular approach for the clinical detection of feline infectious peritonitis. 

The developed LAMP assay can detect FCoV but is not specific for FIPV and therefore cannot be used as a sole means to establish a diagnosis of FIP. Although the results of our RT-LAMP were compared to that of RT-PCR which has been reported to be specific for type II FcoV. It is necessary to use nucleotide sequencing or specific PCR to detect mutations associated with the highly virulent forms of FCoV associated with FIP. Another limitation of this study is that the assay was not run on samples with proven type I infections. To compensate for those limitations, identification of the type of FCoV should be performed. Further studies on the same RT-LAMP means compared with the robust RT-qPCR detecting both type I and type II FCoV are also needed. In this respect, further investigations are required in order to confirm the ability of the RT-LAMP to identify both type I and type II FCoV since the designed primers were based on conserved regions among FCoV, and type I is the predominant FCoV type causing FIP worldwide. Moreover, given the genetic similarity between FIPV and FECV, further investigation is needed on fecal samples that might contain non-virulent FCoV which were not included in this study. In future studies, it would be interesting to see if the LAMP assay could also be used to identify healthy cats that shed FCoV.

## 5. Conclusions

This study describes the first steps toward developing a colorimetric RT-LAMP procedure for detecting FIP-associated FCoV. The novel set of LAMP primer was designed to be highly specific for the FCoV. Compared to conventional PCR, this colorimetric RT-LAMP method has the advantages of sensitivity, convenience, and easy visualization. Hence, this proposed RT-LAMP method can be promising to be further used as applicable diagnostic techniques in conjunction with other molecular approaches for animal and human ongoing pandemic diseases.

## Figures and Tables

**Table 1:** List of primers used in this work for FCoV detection through RT-LAMP assay. Degenerated bases were used according to IUPAC nomenclature: R (A/G), W (A/T), Y (C/T), M (A/C), K (G/T), and H (A/C/T).

Primer Name	Sequence (5′ → 3′)	Ref.
F3	GTACTRCTWCCGTAACGG	This work
B3	CGGGCRAHTTTAAGGTCA	
FIP (F1c-F2)	GACGTAGTGATCCTTACGATYRC-CTATGTYTTCGTGCCTGA	
BIP (B1c-B2)	ACCTGTYCTCMTTACCGAACC-AGAACGCCATTGCAGTTR	
Loop F	TCAACTAGGTCACGACGG	
Loop B	CGTCATGTTGCARGGYTT	
F2	GCTATGTYTTCGTGCCTGA	
B2	AGAACGCCATTGCAGTTR	
F9N	CGTCAACTGGGGAGATGAAC	[18]
R9N	CATCTCAACCTGTGTGTCATC	
F7	TAAAATGGCCKTGGTATGTGT	
R7Sc	ACTTCTCATRAACGGTGC	

**Table 2:** Detection limits of RT-LAMP and conventional RT-PCR.

Detection Method	Detection Limit	
	g/µL	Copy Molecules/µL
NR-based RT-LAMP	20 fg/µL	1.5 × 10^5^
Conventional RT-PCR	20 ng/µL	3.5 × 10^10^

**Table 3:** Comparative analysis of conventional PCR and RT-LAMP for detecting FCoV RNA from body effusion fluid.

RT-LAMP	Conventional RT-PCR			Sensitivity (%) (95% CI)	Specificity (%) (95% CI)	ĸ Value
	Positive	Negative	Total			
Positive	60	1	61	94 (61.1–100.0)	94.12 (55.8–100.0)	0.79
Negative	4	16	20			
Total	64	17	81			

## Data Availability

The data presented in this study are available within the article. Raw data supporting this study are available from the corresponding author.

## Supplementary Materials

The following supporting information can be downloaded at: https://www.mdpi.com/article/10.3390/ani12162075/s1, Figure S1. Gradient temperature optimization of RT-LAMP assay in the range 50–70 °C. Figure S2. Effect of amplification time on RT-LAMP assay. Figure S3. Effect of MgCl_2_ concentrations on RT-LAMP assay. Figure S4. Effect of dNTPs concentrations on RT-LAMP assay. Figure S5. Primer requirement. Table S1: Clinical samples tested with conventional PCR and RT-LAMP using the NR-based method and the agarose gel electrophoresis method.
