# Supplementary material for: Development of Colorimetric Reverse Transcription Loop-Mediated Isothermal Amplification Assay for Detecting Feline Coronavirus
Source: Animals (Basel). 2022 Aug 14;12(16):2075. doi: 10.3390/ani12162075 (PMC9405184; doi:10.3390/ani12162075)
Supplement: Supplementary file 1 [file animals-12-02075-s001.zip › animals-1813811-supplementary.pdf]

animals

MDPI

Supplementary Materials

Development of Colorimetric Reverse Transcription Loop-Mediated Isothermal Amplification Assay for Detecting Feline Coronavirus

Witsanu Rapichai, Wichayet Saejung, Kotchaporn Khumtong, Chaiwat Boonkaewwan, Supansa Tuanthap, Peter A. Lieberzeit, Kiattawee Choowongkomon and Jatuporn Rattanasrisomporn

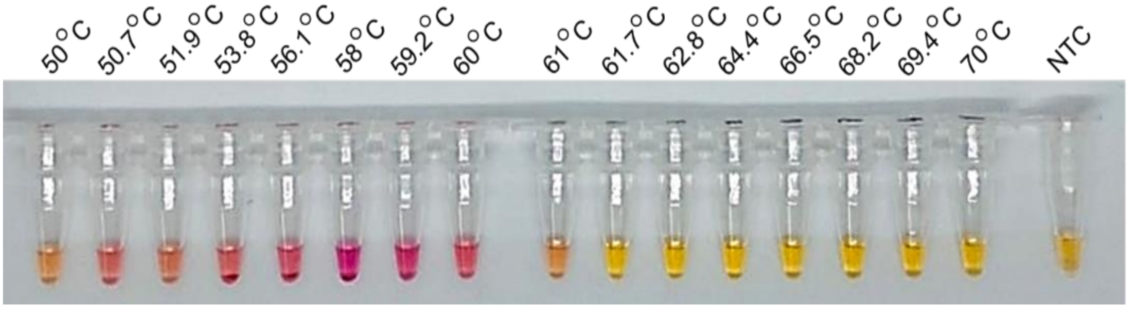

Figure S1. Gradient temperature optimization of RT-LAMP assay in the range 50–70°C. Pink color indicates positive reaction and yellow color indicates negative reaction. NTC, negative control.

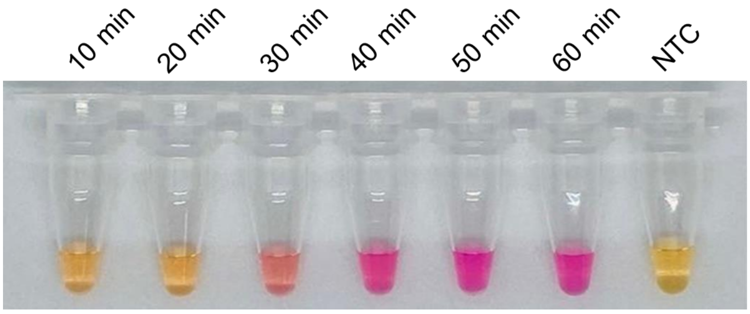

Figure S2. Effect of amplification times on RT-LAMP assay. Pink color indicates positive reaction and yellow color indicates negative reaction. NTC, negative control.

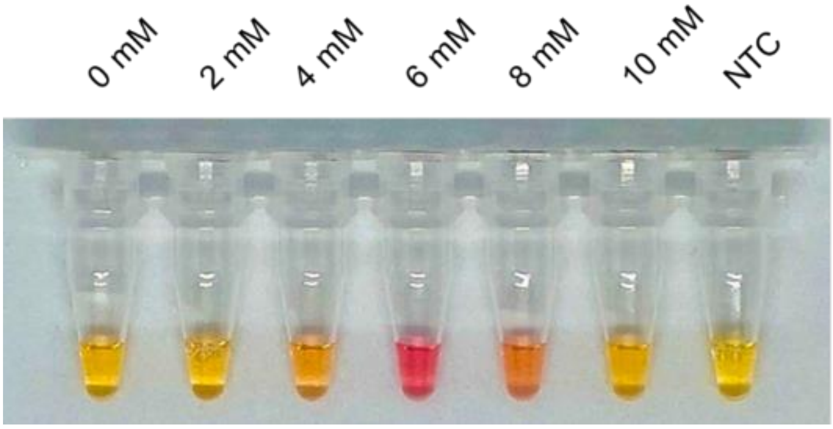

Figure S3. Effect of $MgCl_2$ concentrations on RT-LAMP assay. Pink color indicates positive reaction and yellow color indicates negative reaction. NTC, negative control.

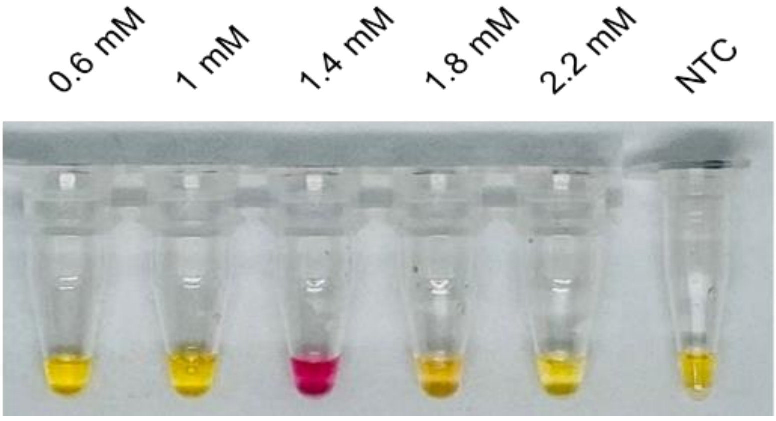

Figure S4. Effect of dNTP concentrations on RT-LAMP assay. Pink color indicates positive reaction and yellow color indicates negative reaction. NTC, negative control.

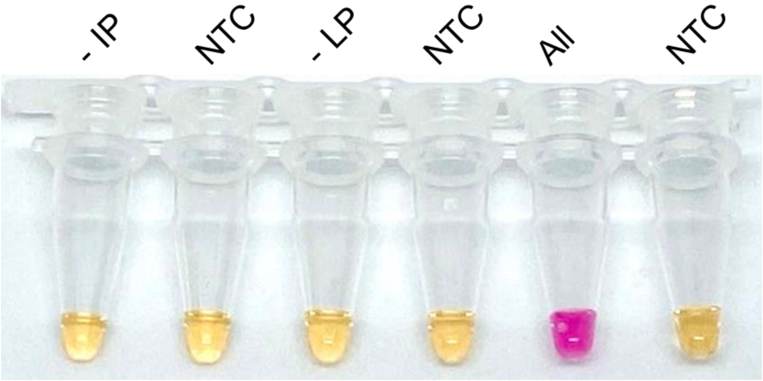

Figure S5. Primer requirement. Pink color indicates positive reaction and yellow color indicates negative reaction. NTC, negative control, IP, inner primers, LP, loop primers, and All, inner-, outer-, and loop primers.

Table S1. Clinical samples were tested with conventional PCR and RT-LAMP using the NR-based method and agarose gel electrophoresis method.

Name	Specimen	PCR F7-R7Sc	RT-LAMP	
			neutral red-based	agarose gel
KU01	body effusion	P	P	P
KU02	body effusion	P	P	P
KU03	body effusion	P	P	P
KU04	body effusion	P	P	P
KU05	body effusion	P	P	P
KU06	body effusion	N	P	P
KU07	body effusion	P	P	P
KU08	body effusion	P	P	P
KU09	body effusion	P	P	P
KU10	body effusion	P	P	P
KU11	body effusion	P	P	P
KU12	body effusion	P	P	P
KU13	body effusion	P	P	P
KU14	body effusion	N	N	N
KU15	body effusion	P	P	P
KU16	body effusion	P	P	P
KU17	body effusion	N	N	N
KU18	body effusion	P	P	P
KU19	body effusion	N	N	N
KU20	body effusion	P	P	P
KU21	body effusion	P	P	P
KU22	body effusion	P	P	P
KU23	body effusion	P	P	P
KU24	body effusion	P	P	P
KU25	body effusion	P	P	P
KU26	body effusion	P	P	P
KU27	body effusion	P	P	P
KU28	body effusion	P	P	P
KU29	body effusion	N	N	N
KU30	body effusion	P	P	P
KU31	body effusion	P	P	P
KU32	body effusion	P	P	P

Name	Specimen	PCR F7-R7Sc	RT-LAMP	
			neutral red-based	agarose gel
KU33	body effusion	P	P	P
KU34	body effusion	P	P	P
KU35	body effusion	P	P	P
KU36	body effusion	N	N	N
KU37	body effusion	P	P	P
KU40	body effusion	P	P	P
KU41	body effusion	P	P	P
KU42	body effusion	P	P	P
KU43	body effusion	P	P	P
KU44	body effusion	P	P	P
KU45	body effusion	P	P	P
KU46	body effusion	P	P	P
KU47	body effusion	P	P	P
KU48	body effusion	P	P	P
KU49	body effusion	P	P	P
KU50	body effusion	P	P	P
KU51	body effusion	P	P	P
KU52	body effusion	N	N	N
KU53	body effusion	P	P	P
KU54	body effusion	P	P	P
KU55	body effusion	P	P	P
KU56	body effusion	N	N	N
KU57	body effusion	P	P	P
KU58	body effusion	P	P	P
KU59	body effusion	P	P	P
KU60	body effusion	P	P	P
KU61/1	body effusion	N	N	N
KU61/2	body effusion	N	N	N
KU61/3	body effusion	N	N	N
KU61/4	body effusion	N	N	N
KU61/5	body effusion	N	N	N
KU62	body effusion	P	P	P
KU64	body effusion	N	N	N
KU66/1	body effusion	P	P	P

Name	Specimen	PCR F7-R7Sc	RT-LAMP	
			neutral red-based	agarose gel
KU66/2	body effusion	P	P	P
KU66/3	body effusion	P	P	P
KU67	body effusion	N	N	N
KU68	body effusion	P	P	P
KU71	body effusion	P	P	P
KU72	body effusion	P	P	P
KU73	body effusion	P	P	P
KU74	body effusion	N	N	N
KU75	body effusion	P	P	P
KU76	body effusion	P	N	N
KU77	body effusion	N	N	N
KU78	body effusion	P	P	P
KU79	body effusion	P	N	N
KU80	body effusion	P	N	N
KU81	body effusion	P	N	N
